# Proteomic profiling of *Escherichia coli* cytoplasmic proteins under sublethal boric acid stress

**DOI:** 10.55730/1300-0152.2745

**Published:** 2025-04-21

**Authors:** Bekir ÇÖL, Begüm Hazar ÇİFTÇİ, Merve SEZER KÜRKÇÜ, Esra DİBEK

**Affiliations:** 1 Department of Biology, Faculty of Science, Muğla Sıtkı Koçman University, Muğla, Turkiye; 2 Biotechnology Research Center, Muğla Sıtkı Koçman University, Muğla, Turkiye; 3 Research and Application Center for Research Laboratories, Muğla Sıtkı Koçman University, Muğla, Turkiye; 4 Department of Pharmacy Services, Köyceğiz Vocational School of Health Services, Muğla Sıtkı Koçman University, Muğla, Turkiye

**Keywords:** Boron stress, bacterial proteomics, SodA, KduD, metabolic adaptation

## Abstract

**Background/aim:**

Boron is an essential micronutrient for plants and certain bacteria, where it plays critical roles in cellular processes at low concentrations. However, elevated levels of boron-containing compounds, such as boric acid, exhibit antimicrobial toxicity. Although the physiological effects of boric acid on bacteria have been partially characterized, its proteome-wide impacts remain poorly elucidated. This study employs a 2D-PAGE-based proteomic approach to investigate how sublethal boric acid stress alters the cytoplasmic proteome of *Escherichia coli* BW25113.

**Materials and methods:**

*E. coli* BW25113 cultures were grown to mid-log phase in tryptic soy broth (TSB) and exposed to 70 mM boric acid (a sublethal concentration) or left untreated as a control. Cytoplasmic protein extracts were subjected to 2D-PAGE analysis to identify differentially expressed proteins. Selected protein spots were excised, identified via MALDI-TOF mass spectrometry, and validated by RT-PCR to assess corresponding mRNA expression levels.

**Results:**

Proteomic analysis revealed 12 differentially regulated cytoplasmic proteins under boric acid stress. Upregulated proteins included SodA, KduD, KduI, DeoB, Icd, AceE, RpsM, TdcE, Tuf1, LexA, and LamB, while GatY was downregulated. Functional annotation linked these proteins to oxidative stress defense (SodA), carbohydrate metabolism (KduD, KduI, DeoB), energy production (Icd, AceE), translation (RpsM, Tuf1), and membrane integrity (LamB). RT-PCR validation confirmed transcriptional upregulation of *sodA*, *kduD*, and *kduI*, corroborating proteomic findings. These results suggest that boric acid disrupts metabolic homeostasis, induces oxidative stress, and modulates structural and translational processes in *E. coli*.

**Conclusion:**

This study provides the first proteomic evidence of *E. coli*’s cytoplasmic response to boric acid stress, highlighting its multifaceted effects on metabolic, oxidative, and translational pathways. The upregulation of KduI and KduD, enzymes involved in carbohydrate utilization, points to potential adaptive mechanisms for boron detoxification. Further investigation into these targets could elucidate molecular strategies for bacterial boron tolerance and inform the development of boron-based antimicrobials.

## 1. Introduction

Boron, a metalloid with unique electron-deficient properties, is an essential micronutrient for plants, some animals, and select microorganisms, where it stabilizes biomolecules through reversible diester bonds with *cis*-diol groups (e.g., cell wall polysaccharides, ribose in RNA) (Uluisik et al., 2018). Its critical role in plant physiology was first established by Warington (1923), who linked boron deficiency to growth arrest and structural deformities (Warington, 1923). Beyond plants, boron’s biological significance extends to animals, where it modulates immune function and bone metabolism (Pizzorno, 2015), and to bacteria, where it contributes to biofilm formation and antibiotic biosynthesis (Schummer et al., 1995; Chen et al., 2022). Interestingly, boron compounds can also influence bacterial quorum sensing, which is crucial for biofilm development and virulence (Konaklieva and Plotkin, 2024). However, boron exhibits a narrow concentration window between essentiality and toxicity: while low doses support growth in boron-dependent organisms like Cyanobacteria (Bonilla et al., 1990), elevated levels disrupt mitosis in eukaryotic cells (Hilal et al., 2024) and inhibit bacterial proliferation (Dibek et al., 2020; Çöl et al., 2024), underscoring its dual role as a micronutrient and antimicrobial agent. This toxicity can be exploited for antibacterial applications, particularly in environments where boron concentrations may fluctuate (Ahmed and Fujiwara, 2010; Celebi et al., 2024).

The molecular mechanisms underlying boron toxicity has not been comprehensively studied, particularly in prokaryotes. In plants, boron toxicity destabilizes cell wall pectin networks (Funakawa and Miwa, 2015), while in bacteria, it is hypothesized to interfere with ribose-dependent processes (e.g., RNA metabolism, quorum signaling) (Ahmed and Fujiwara, 2010). Studies have highlighted boron’s potential as a therapeutic agent, demonstrating its anti-tumor activity through mitotic disruption and its role in boron-containing antibiotics like tartrolons and epetraborole (Barranco and Eckhert, 2004; Dibek et al., 2020; Babayeva et al., 2024). Recent genomic studies in *E. coli* have started to identify genes involved in intrinsic boric acid resistance, highlighting the complexity of bacterial responses (Çöl et al., 2024). While boron toxicity mechanisms are partially understood, studies investigating bacterial responses to boron stress at the proteomic level are still limited (Sen et al., 2023). To address this gap, we selected *E. coli* BW25113, a model strain with unparalleled genetic tractability as the parent of the Keio knockout collection, for proteomic profiling under sublethal boric acid stress.

Proteomic approaches are uniquely suited to unravel dynamic cellular responses to environmental stressors like boron. Unlike static genomic analyses, proteomics captures post-translational modifications, protein turnover, and stress-induced expression shifts (Cho, 2007). For instance, 2D-PAGE has resolved metal-stress responses in *E. coli*, revealing upregulated peroxidases and chaperones under cadmium exposure (Han and Lee, 2006). However, no studies to date have applied this methodology to boron stress on *E. coli*, leaving a critical gap in our knowledge of bacterial metalloid resistance. Understanding the proteomic response to boric acid is crucial for developing effective boron-based antimicrobial strategies and for comprehending bacterial adaptation in boron-rich environments (Sen et al., 2023).

In this study, we investigated the cytoplasmic proteome of *E. coli* BW25113, a model organism with unparalleled genetic and biochemical tractability (Blount, 2015), under sublethal boric acid stress. Using 2D-PAGE coupled with MALDI-TOF mass spectrometry, we identify 12 differentially expressed proteins involved in oxidative defense, carbohydrate metabolism, and translational fidelity. RT-PCR validation confirms transcriptional upregulation of key targets, including *sodA* (superoxide dismutase) and *kduI* (4-deoxy-L-threo-5-hexosulose-uronate ketol-isomerase). Our findings provide the first proteomic evidence of *E. coli*’s adaptive response to boron, offering insights into its dual role as a micronutrient and antimicrobial agent.

## 2. Materials and methods

*E. coli* strain BW25113 was used in this study. Acrylamide, N,N′-methylene bis-acrylamide, ammonium persulfate (APS), dithiothreitol (DTT), ampholyte, 3-[(3-cholamidopropyl)dimethylammonio]-1-propanesulfonate (CHAPS), tributylphosphine (TBP), 7 cm pH 4–7 IPG ReadyStrip, mineral oil, and iodoacetamide were purchased from Bio-Rad Laboratories (Hercules, CA, USA). Sodium dodecyl sulfate (SDS), urea, thiourea, TEMED, tris, ethylenediaminetetraacetic acid (EDTA), protease inhibitor cocktail, trichloroacetic acid (TCA), acetone, glycerol, and glycine were purchased from Sigma-Aldrich (USA). Colloidal Coomassie G-250 stain (Bloo Moose staining solution) was purchased from KeraFAST (USA).

### 2.1. Bacterial strain and growth conditions

**Figure 1:**
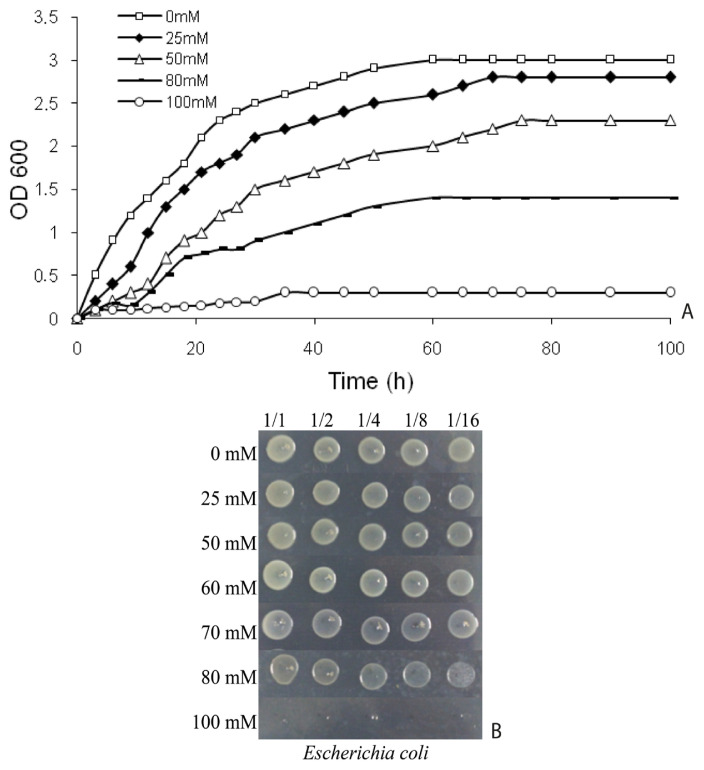
Time-dependent growth curves (A) and spot assays (B) illustrating growth inhibition and viability of *E. coli* BW25113 under boric acid stress.

*E. coli* BW25113 was cultured in tryptic soy broth (TSB; BD Biosciences) at 30 °C with shaking (150 rpm). To determine lethal and sublethal boric acid concentrations, growth curves were complemented by spot assays according to established protocols (Çöl et al., 2024). Growth curves were generated in TSB supplemented with 0, 25, 50, 80, and 100 mM boric acid (H_3_BO_3_; Sigma-Aldrich), while spot tests on TSA plates identified complete growth inhibition thresholds. Optical density (OD_600_) was measured hourly for several days using a spectrophotometer (Optizen, South Korea), with colony viability simultaneously monitored through spot analyses (Figure 1). The sublethal concentration (70 mM boric acid) was operationally defined as the highest dose permitting ≥50% viability relative to untreated controls, as validated by both growth curve interpolation and spot test quantitation (Figure 1).

### 2.2. Protein extraction

Mid-log-phase cultures (OD_600_ = 0.5 ± 0.05) were treated with 70 mM boric acid or left untreated for 1 h. Cells were harvested by centrifugation (10,000 × g, 10 min, 4 °C; Thermo Scientific SL16R), washed thrice with ice-cold phosphate-buffered saline (PBS; pH 7.4), and lysed in 2D rehydration buffer (8 M urea, 2 M thiourea, 2% CHAPS, 50 mM DTT, 0.5% ampholyte pH 3 -10, 1X protease inhibitor cocktail). Lysates were sonicated (5 cycles: 20 s pulse, 40 s rest; Bandelin Sonicator) and clarified by centrifugation (18,000 × g, 10 min, 4 °C). Proteins were precipitated using 10% trichloroacetic acid (TCA)/90% acetone (−20 °C, 2 h), washed with ice-cold acetone, and resuspended in rehydration buffer. Protein concentration was quantified via Bradford assay (Bio-Rad), with bovine serum albumin (BSA) as a standard. It should be noted that while our extraction protocol targeted cytoplasmic proteins, the use of urea and CHAPS may have solubilized some membrane-associated proteins, a limitation inherent to 2D-PAGE.

### 2.3. Two-dimensional gel electrophoresis

Isoelectric focusing (IEF) was performed using 7 cm pH 4–7 immobilized pH gradient (IPG) strips (Bio-Rad). Strips were passively rehydrated (12 h, 20 °C) with 400 μg protein lysate in rehydration buffer containing 1% tributylphosphine (TBP) and 1% ampholyte. IEF conditions: 250 V (20 min), linear ramp to 4.000 V (2 h 50 min), and 40.000 V·h rapid focusing (Protean IEF Cell, Bio-Rad). Strips were equilibrated sequentially in buffer I (6 M urea, 0.375 M Tris-HCl pH 8.8, 2% SDS, 20% glycerol, 2% DTT) and buffer II (6 M urea, 0.375 M Tris-HCl pH 8.8, 2% SDS, 20% glycerol, 2.5% iodoacetamide).

Second-dimension SDS-PAGE was performed on 12% polyacrylamide gels (Mini-PROTEAN® Tetra Cell, Bio-Rad) at 180 V for 55 min. Gels were stained with colloidal Coomassie G-250 (KeraFAST, USA) and imaged (VersaDoc™ 4000 MP, Bio-Rad).

### 2.4. Image analysis and protein identification

Protein spot detection, matching, and intensity quantification were performed using PDQuest Advanced Software (v8.0.1, Bio-Rad), as described by (Bal Albayrak et al., 2025). Raw spot intensities were normalized against the total density of valid spots on each gel to minimize technical variability, followed by uniform background subtraction. Spots exhibiting a ≥2.0-fold change (Student’s t-test, p < 0.05) between control and treated groups were identified as statistically significant. Triplicate gels (n = 3) were analyzed to ensure reproducibility. The selected spots were excised (ExQuest Spot Cutter, Bio-Rad), destained, and digested with trypsin (25 ng/μL; 37 °C, overnight) (Bal Albayrak et al., 2025). Peptides were eluted with 0.1% trifluoroacetic acid (TFA), mixed with α-cyano-4-hydroxycinnamic acid (CHCA) matrix, and analyzed by MALDI-TOF/TOF (AB SCIEX TOF/TOF 5800 instrument, Framingham, MA, USA). Peptide mass fingerprints were analyzed using Mascot Server v2.6 (Matrix Science) against the NCBI *E. coli* database (TaxID: 562) with a mass tolerance of ±0.2 Da and significance thresholds of p < 0.05 and Mascot score >40.

### 2.5. RT-PCR validation

Total RNA was isolated (RNeasy Mini Kit, Qiagen) from treated/untreated cells, treated with DNase I (Ambion), and reverse-transcribed (RevertAid First Strand cDNA Synthesis Kit, Thermo Scientific). Gene-specific primers for six genes (*sodA, kduD, kduI, icd, deoB* and including the control 16S) were designed (Table 1), and RT-PCR was performed (GoTaq® Green Master Mix, Promega) under the following conditions: 95 °C (2 min); 30 cycles of 95 °C (30 s), 55 °C (30 s), 72 °C (30 s); 72 °C (5 min). Products were resolved on 1.5% agarose gels and quantified (Bio-Rad). Band intensities were quantified using ImageJ densitometry (National Institutes of Health, USA), normalized to the 16S rRNA internal control, and expressed as fold-change relative to untreated controls. Error bars represent the standard deviation (SD) of three independent biological replicates.

## 3. Results

### 3.1. Boric acid inhibits *E. coli* growth in a dose-dependent manner

Growth curve analyses revealed significant growth inhibition at ≥50 mM boric acid, with 100 mM causing near-complete suppression (Figure 1A). Bacterial growth was monitored in TSB medium supplemented with 0, 25, 50, 80, and 100 mM boric acid at 30 °C with shaking (150 rpm). Optical density (OD_600_) was measured hourly for several days. Spot tests on TSA plates (Çöl et al., 2024) confirmed dose-dependent reductions in colony-forming ability, with no viable colonies observed at ≥80 mM after 24-h exposure (Figure 1B). A sublethal concentration of 70 mM was selected based on interpolation of growth curves, representing ~50% inhibition (IC_50_) to ensure sublethal stress conditions while eliciting measurable proteomic responses. The interpolated IC_50_ value correlated strongly with spot test viability indices, validating the dual-method approach for threshold determination.

### 3.2. Proteomic profiling identifies 12 differentially expressed proteins

**Figure 2:**
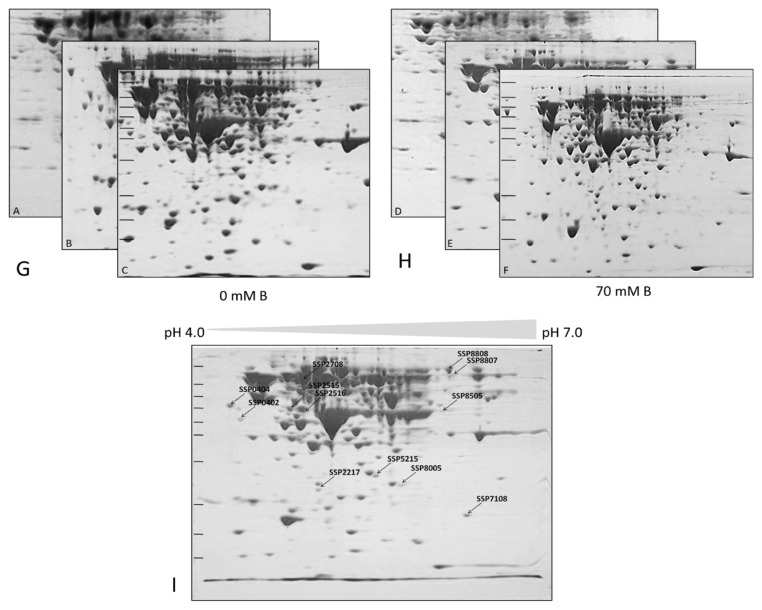
Two-dimensional gel electrophoresis (2D-PAGE) analysis of *E. coli* BW25113 cytoplasmic proteins under boric acid stress.

Comparative 2D-PAGE analysis detected 12 cytoplasmic proteins with significant expression changes (≥2-fold, p < 0.05) under boric acid stress (Figure 2). MALDI-TOF analysis identified these proteins as SodA, KduD, KduI, DeoB, Icd, AceE, RpsM, TdcE, Tuf1, LexA, LamB (upregulated), and GatY (downregulated) (Table 2). Figure 2 illustrates representative 2D-PAGE gels of cytoplasmic proteins from untreated *E. coli* BW25113 cultures (control) (Figure 2A–C) and corresponding gels from cultures treated with 70 mM boric acid (70 mM B) (Figures 2D–2F). Proteins were separated by isoelectric focusing (IEF) using 7 cm pH 4–7 IPG strips, followed by SDS-PAGE on 12% polyacrylamide gels. Gels were stained with colloidal Coomassie G-250 (Kerafast). Biological triplicates are shown (n = 3). (Figure 2G) and (Figure 2H) indicate the three 2D gels (triplicate experimental results) for the control and treated samples. PDQuest 8.0.1 analysis of differentially expressed protein spots, assigned unique Standard Spot Numbers (SSP; e.g., SSP 2515, SSP 2516) (Figure 2I). Spots showing ≥2-fold intensity change (p < 0.05, Student’s t-test) between control and treated groups are labeled. Molecular weight markers (kDa) and isoelectric point (pI) ranges are indicated.

Protein spots were analyzed using PDQuest 8.0.1 software to generate reference gels (master gels) and quantify differential expression. Following spot selection, excised gel fragments underwent in-gel tryptic digestion. Peptide mass fingerprinting via MALDI-TOF matched spectra to the NCBI *E. coli* database, with stringent statistical validation. Mascot scores, calculated as −10·log(P), where p represents the probability of random peptide matches, exceeded significance thresholds (p < 0.05). Differential expression was further confirmed by Student’s t-test, comparing normalized spot volume means (%Vol) between treated (P) and control (C) groups (Table 2).

Differentially regulated proteins upon boric acid stress encode the activities as follows: SodA (superoxide dismutase [Mn]), KduD (2-dehydro-3-deoxy-D-gluconate 5-dehydrogenase), KduI (4-deoxy-L-threo-5-hexosulose-uronate ketol-isomerase), DeoB (phosphopentomutase), Icd (isocitrate dehydrogenase [NADP]), AceE (pyruvate dehydrogenase E1 component), RpsM (30S ribosomal protein S13), TdcE (keto-acid formate acetyltransferase), Tuf1 (elongation factor Tu), LexA (LexA repressor), LamB (maltoporin), and GatY (D-tagatose-1,6-bisphosphate aldolase subunit; downregulated). Strong concordance was observed between theoretical and experimental molecular weights (average error: ±5%) and isoelectric points (average pI error: ±0.3), validating the reliability of identifications (Table 2).

### 3.3. RT-PCR confirms transcriptional upregulation of key targets

**Figure 3:**
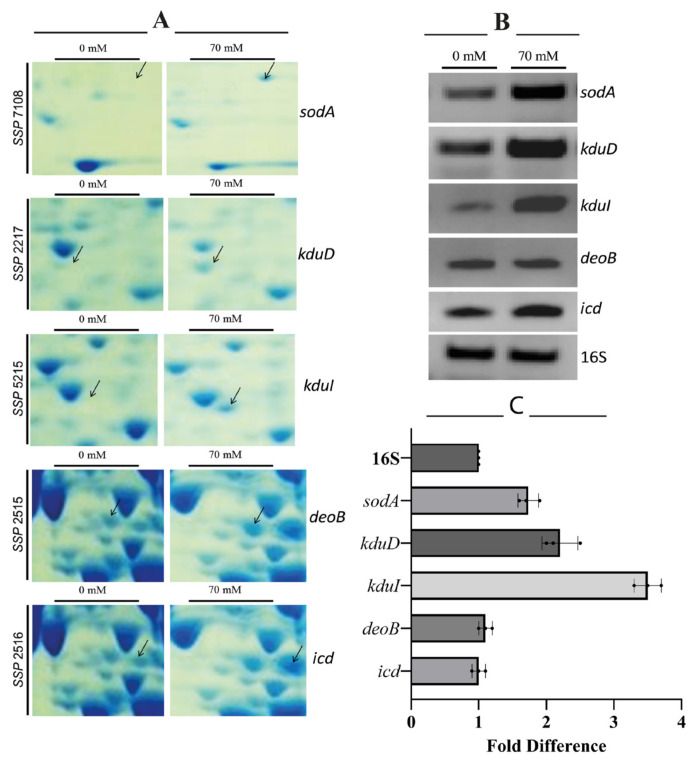
Proteomic analysis and RT-PCR validation of boric acid-induced protein upregulation in *E. coli* BW25113

**Figure 4:**
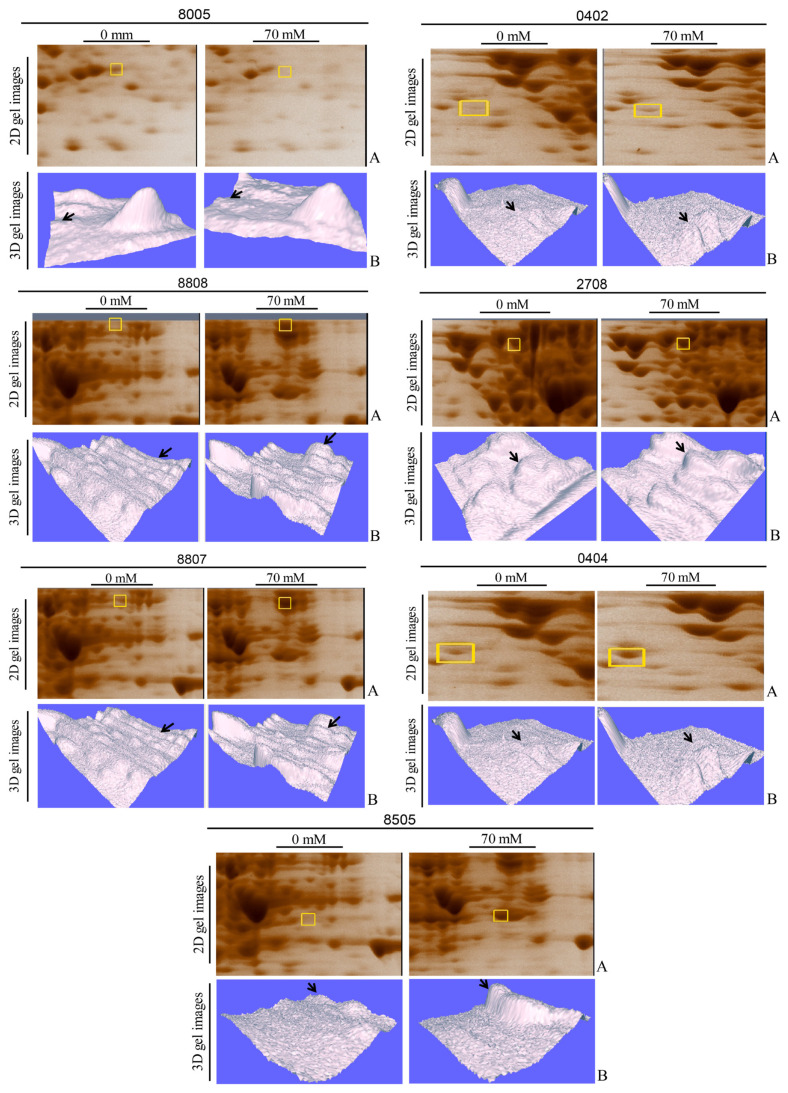
Differential expression of cytoplasmic proteins in *E. coli* BW25113 under boric acid stress.

The primers designed for the genes are given in Table 1. RT-PCR validated increased mRNA levels for *sodA*, *kduD*, *kduI*, *deoB*, and *icd* (Figure 3), correlating with proteomic data (p<0.01). In Figure 3A, enlarged segments of 2D-PAGE gels highlighting differentially expressed protein spots under 70 mM boric acid stress are shown. Labeled spots correspond to SodA (superoxide dismutase, SSP 7108), KduD (2-dehydro-3-deoxy-D-gluconate 5-dehydrogenase, SSP 2217), KduI (4-deoxy-L-threo-5-hexosulose-uronate ketol-isomerase, SSP 5215), DeoB (phosphopentomutase, SSP 2515), and Icd (isocitrate dehydrogenase, SSP 2518). Figure 3B illustrates RT-PCR analysis of corresponding mRNA levels for *sodA*, *kduD*, *kduI*, *deoB*, and *icd*. Total RNA was isolated from untreated (−) and 70 mM boric acid-treated (+) cultures. 16SrRNA gene served as a housekeeping control. Band intensities show relative differential gene expression levels. Expression levels of *kduD*, *kduI*, and *sodA* genes were significantly upregulated in the boric acid-treated samples (Figure 3C). Differential expression patterns for the proteins were visualized using 3D gel images and are shown in Figure 4, where segmented 2D-PAGE gel images highlighting protein spots with significant expression changes (Figure 4A) are included. Labeled spots correspond to SSP 8005, SSP 0402, SSP 8808, SSP 2708, SSP 8807, SSP 0404, and SSP 8505 (Figure 4B). Three-dimensional (3D) intensity profiles of selected protein spots were generated using PDQuest 8.0.1 software. Spot volumes were normalized to total gel density, with untreated controls compared to 70 mM boric acid-treated samples. Arrows indicate ≥2-fold intensity changes.

## 4. Discussion

This study demonstrates that exposure of *E. coli* BW25113 to boron (boric acid) induces differential expression of cytoplasmic proteins, reflecting adaptive responses to the metalloid stress. Functional annotation using KEGG, UniProt, and EcoCyc databases linked these proteins to critical pathways, including energy metabolism, carbohydrate utilization, oxidative stress defense, and translation (Table 3). Below, we interpret their roles in boron-related regulatory mechanisms and propose broader implications for bacterial stress adaptation.

### 4.1. Energy and carbohydrate metabolism

Key enzymes in energy metabolism, such as phosphopentomutase (DeoB) and isocitrate dehydrogenase (Icd), were upregulated under boric acid stress. DeoB, a nucleoside salvage pathway enzyme, has been implicated in stress responses in *Lactococcus lactis* and *Streptococcus mutans* during nutrient deprivation (Duwat et al., 1999; Xue et al., 2010). Our findings extend this role to boron stress, suggesting DeoB aids in maintaining nucleotide pools under metabolic disruption. In *E. coli*, DeoB is also important for deoxyribonucleoside metabolism, which is essential for DNA replication and repair under stress conditions (Valle et al., 2015). Similarly, Icd, a TCA cycle enzyme regulated via phosphorylation (Link et al., 1997), supports NADPH production, which is critical for countering oxidative damage (Krisko et al., 2014). Its upregulation aligns with boron’s reported disruption of redox balance in yeast (Uluisik et al., 2018), pointing to conserved stress-mitigation strategies. Carbohydrate metabolism was further impacted by the downregulation of GatY (D-tagatose-1,6-bisphosphate aldolase) and upregulation of KduI/KduD, enzymes involved in glucuronate/galacturonate catabolism. KduI/KduD are known to mediate osmotic stress adaptation (Rothe et al., 2013), and their induction here suggests boric acid mimics osmotic shock, driving *E. coli* to scavenge alternative carbon sources. In *E. coli*, KduI and KduD are part of the hexuronate utilization pathway, which allows bacteria to utilize plant-derived sugar acids as carbon sources, particularly under stress conditions (Rothe et al., 2013).

### 4.2. Oxidative stress and DNA repair

SodA (Mn-superoxide dismutase) and LexA (SOS response repressor) were significantly upregulated in response to boron stress. SodA neutralizes superoxide radicals (O_2_^−^) generated during oxidative stress, a process typically regulated by the SoxRS system (Britton and Fridovich, 1977). Its induction implies boric acid disrupts redox homeostasis, likely through indirect ROS generation, as observed in various biological systems (Carlioz and Touati, 1986; Hopkin et al., 1992; Iuchi and Weiner, 1996). SodA is a key antioxidant enzyme in *E. coli*, and its upregulation is a hallmark of oxidative stress response induced by various agents, including metals and antibiotics (Tawiah et al., 2025). LexA, which represses DNA repair genes until activated by RecA (Fernández De Henestrosa et al., 2000), suggests boron induces subtle genotoxic stress, though further assays (e.g., comet assays) are needed to confirm DNA damage. The SOS response, regulated by LexA, is a global stress response in *E. coli* activated by DNA damage, and its induction suggests that boric acid may indirectly cause DNA lesions or replication stress (Fernández De Henestrosa et al., 2000; Jones and Uphoff, 2021).

### 4.3. Translational machinery

Essential translation components, including Tuf1 (elongation factor Tu) and RpsM (30S ribosomal protein S13) (Weijland et al., 1992; Schuwirth et al., 2005) were upregulated despite metabolic stress. Tuf1, critical for tRNA delivery to ribosomes, is often growth rate-dependent, while a strain with a *rpsM* deletion exhibits a significant growth defect (Cukras and Green, 2005). Their sustained expression underscores *E. coli*’s prioritization of protein synthesis under stress. Upregulation of translation factors like Tuf1 under stress can be part of a general stress response to remodel the proteome and prioritize synthesis of stress-protective proteins (Segura et al., 2005). RpsM, as a ribosomal protein, might be upregulated to maintain ribosome biogenesis or stability under boric acid stress, ensuring efficient protein synthesis (van Vliet et al., 2018).

### 4.4. Limitations and future directions

While this study advances our understanding of *E. coli*’s adaptation to boric acid, the inherent limitations of 2D-PAGE must be acknowledged. This technique is biased toward abundant, soluble proteins and may underrepresent low-abundance or membrane-associated targets. Integrating complementary approaches such as LC-MS/MS could mitigate this constraint and provide a more comprehensive proteomic profile. Our findings establish three novel adaptive strategies employed by *E. coli* under boron stress: oxidative stress mitigation via SodA upregulation, metabolic flexibility through Icd-mediated NADPH production and DeoB-driven nucleoside salvage, and carbohydrate metabolism rewiring involving KduI/KduD activation (Table 3).

To build on these insights, future work could prioritize functional validation through targeted gene knockouts (e.g., *ΔsodA*, *ΔkduD*) to confirm the roles of identified proteins in boron tolerance. Regulatory exploration via transcriptomic profiling may uncover transcriptional networks governing boron-responsive pathways, including potential transcription factors. Comparative analyses in pathogens like *Salmonella* or *Pseudomonas aeruginosa* could identify conserved or unique resistance mechanisms with antimicrobial implications. Additionally, evaluating boron-containing compounds as antibiotic adjuvants might offer new strategies to counteract multidrug-resistant pathogens. Notably, recent studies leveraging boron-tolerant strains like *Pseudomonas sp.* BC4B in Fe-MOF bioanodes demonstrate the potential to harness microbial adaptations for bioelectrochemical technologies, bridging fundamental research with sustainable energy applications (Avcı et al., 2023).

This work underscores boron’s dual role as both a micronutrient and a stress inducer while providing molecular insights into bacterial survival strategies. Further elucidation of these mechanisms could advance diverse fields, from designing boron-based antimicrobials to engineering robust microbial systems for bioremediation or bioenergy production in boron-rich environments.

## Supplementary material

Figure S Comparative bar graph of selected cytoplasmic proteins showing differential expression in *E. coli* BW25113 under boric acid stress. Bars represent the mean normalized spot volumes (%Vol) of proteins from control (0 mM boric acid, black) and 70 mM boric acid-treated (grey) cultures. Error bars indicate standard deviation (n *=* 3). The X-axis displays the Standard Spot Numbers (SSP) and protein names of the differentially expressed proteins. The Y-axis represents the relative value of the normalized spot volume (%Vol). This graph was generated using the ImageJ program based on data obtained from 2D-PAGE gels analyzed with PDQuest 8.0.1 software.

## Figures and Tables

**Table 1:** Primers used in this study for RT-PCR analysis.

Primer	Sequence (5′ → 3′)
16SrRNA-F	CCGTGTCTCAGTTCCAGT
16SrRNA-R	TGAGCCTAGGTCGGATTA
SodA-F	GGAAATCCACCACACCAAAC
SodA-R	GATAGCCGCTTTCAGGTCAC
DeoB-F	AAGAAACCTTTGGCCTGGAT
DeoB-R	ACCGACAGAAACCACCTGAC
Icd-F	ATATGCCGGTCAGGACAAAG
Icd-R	GCGTCACCAAACTCTGAACA
KduD-F	AACCGACTGAAACCATCGAG
KduD-R	ATCAATCCGGCGTTATTCAC
KduI-F	GGTGCCGGTACGATTACTGT
KduI-R	CGACGGTTACTGGTGAGGTT

**Table 2:** Identification of proteins in *E. coli* BW25113 strain using Mascot Analysis.

SSP NO	% sequence coverage	Mascot score/appraisement (p)	pI	Theoretical Protein MW, Da	Experimental Protein MW, Da	Identified Protein (gene)	Regulation^a^
2515	66%	670/3.2e-062	5.11	44342	47,000	Phosphopentomutase, *deoB*	Up
2516	46%	673/1.6e-062	5.15	45727	47,000	Isocitrate dehydrogenase [NADP], *icd*	Up
2217	59%	566/8.1e-052	5.24	27053	28,000	2-dehydro-3-deoxy-D-gluconate-5-dehydrogenase, *kduD*	Up
5215	50%	596/8.1e-055	5.70	31056	30,000	4-deoxy-L-threo-5-hexosulose-uronate ketol-isomerase, *kduI*	Up
7108	68%	555/1e-050	6.45	23065	24,000	Superoxide dismutase [Mn], *sodA*	Up
8005	27%	401/2.6e-035	5.87	30766	24,000	D-tagatose-1,6-bisphosphate aldolase subunit, *gatY*	Down
8808	12%	83/0.0015	5.46	99606	50,000	Pyruvate dehydrogenase E1 component, *aceE*	Up
8807	13%	46/9.1	11.45	13539	47,000	30S ribosomal protein S13, *rpsM*	Up
0402	11%	165/1e-011	5.48	85881	32,000	Keto-acid formate acetyltransferase, *tdcE*	Up
2708	42%	664/1.3e-061	5.3	43256	43,000	Elongation factor Tu 1, *tuf1*	Up
0404	13%	42/21	5.33	22654	36,000	LexA repressor, *lexA*	Up
8505	70%	89/1.3e-078	5.35	49820	24,000	Maltoporin, *lamB*	Up

a Quantitative protein expression changes under boric acid stress. Fold differences (70 mM vs. untreated control) were determined by ImageJ densitometry analysis of 2D-PAGE spot intensities. Normalized spot intensities were plotted as mean ± SEM (see Supplementary Figure S).

**Table 3:** Functional evaluation of differentially expressed proteins in response to boric acid stress in *E. coli*.

Activity/Function	Protein	Pathway/Process	Role and Regulation
Energy metabolism	Phosphopentomutase (DeoB)	Pentose phosphate pathway, nucleotide biosynthesis	Catalyzes phosphorylation shifts for ribose transformation (Duwat et al., 1999; Xue et al., 2010); may be influenced by boric acid in energy flow.
	Isocitrate dehydrogenase (Icd)	Citric acid cycle	Converts isocitrate to α-ketoglutarate in the citric acid cycle; regulated by phosphorylation. Produces NADPH for energy and oxidative stress management (Walsh and Koshland 1985; Link et al., 1997; Krisko et al., 2014) upregulated by boric acid. Upregulation suggests a response to oxidative stress potentially induced by boron exposure.
	Pyruvate dehydrogenase E1 (AceE)	Glycolysis to TCA transition	Converts pyruvate to acetyl-CoA for energy production (KEGG, Uniprot); may have been upregulated by boron during energy demands.
	Keto-acid formate acetyltransferase (TdcE)	Pyruvate metabolism	Converts pyruvate for anaerobic metabolism (Hagewood et al., 1994; Sawers, 2001); EcoCyc, KEGG, Uniprot); boron may induce metabolic shifts.
Carbohydrate metabolism	D-tagatose-1,6-bisphosphate aldolase (GatY)	Galactose metabolism	Breaks down galactose derivatives (UniProt; Brinkkötter et al., 2002) boron may modulate sugar acid metabolism, emphasizing its role in carbon flux.
	4-deoxy-L-threo-5-hexosulose-uronate ketol-isomerase (KduI)	Sugar acid metabolism	Isomerization of sugar acids in glucuronate/galacturonate metabolism, converts sugar derivatives to central metabolites (Rothe et al., 2013), highlights boron’s role in osmotic and stress adaptation processes.
	2-dehydro-3-deoxy-D-gluconate-5-dehydrogenase (KduD)	D-gluconate and pentose phosphate pathway	Regulates sugar acid flux (Rothe et al., 2013) boron may affect some intermediate enzymatic activities such as this one.
	Maltoporin (LamB)	Carbohydrate transport	Transports maltose and related sugars (Benz et al., 1987; KEGG, Uniprot) regulated by boron due to altered carbohydrate needs or boron may bind to some sugars.
Stress response	Superoxide dismutase (SodA)	Oxidative stress response	Detoxifies reactive oxygen species, may play a key role in oxidative stress defense under boric acid-induced stress (Britton and Fridovich 1977; Carlioz and Touati 1986; Hopkin et al., 1992; Iuchi and Weiner 1996) boron may enhance its expression by causing oxidative stress.
	LexA repressor (LexA)	SOS response, DNA repair	Suppresses DNA repair genes unless damaged (Fernández De Henestrosa et al., 2000; McKenzie et al., 2022) boron may indirectly trigger SOS response.
Protein synthesis and translation	Elongation factor Tu 1 (Tuf1)	Protein synthesis	Aids tRNA binding during protein elongation (KEGG, UNIPROT) (Weijland et al., 1992).
	30S ribosomal protein S13 (RpsM)	Translation	Ribosomal component for protein synthesis (Schuwirth et al., 2005) boron may affect translation directly or indirectly.
