# Supplementary figures and images for: Proteomic profiling of Escherichia coli cytoplasmic proteins under sublethal boric acid stress
Source: Turk J Biol. 2025 Apr 21;49(3):280–91. doi: 10.55730/1300-0152.2745 (PMC12266352; doi:10.55730/1300-0152.2745)

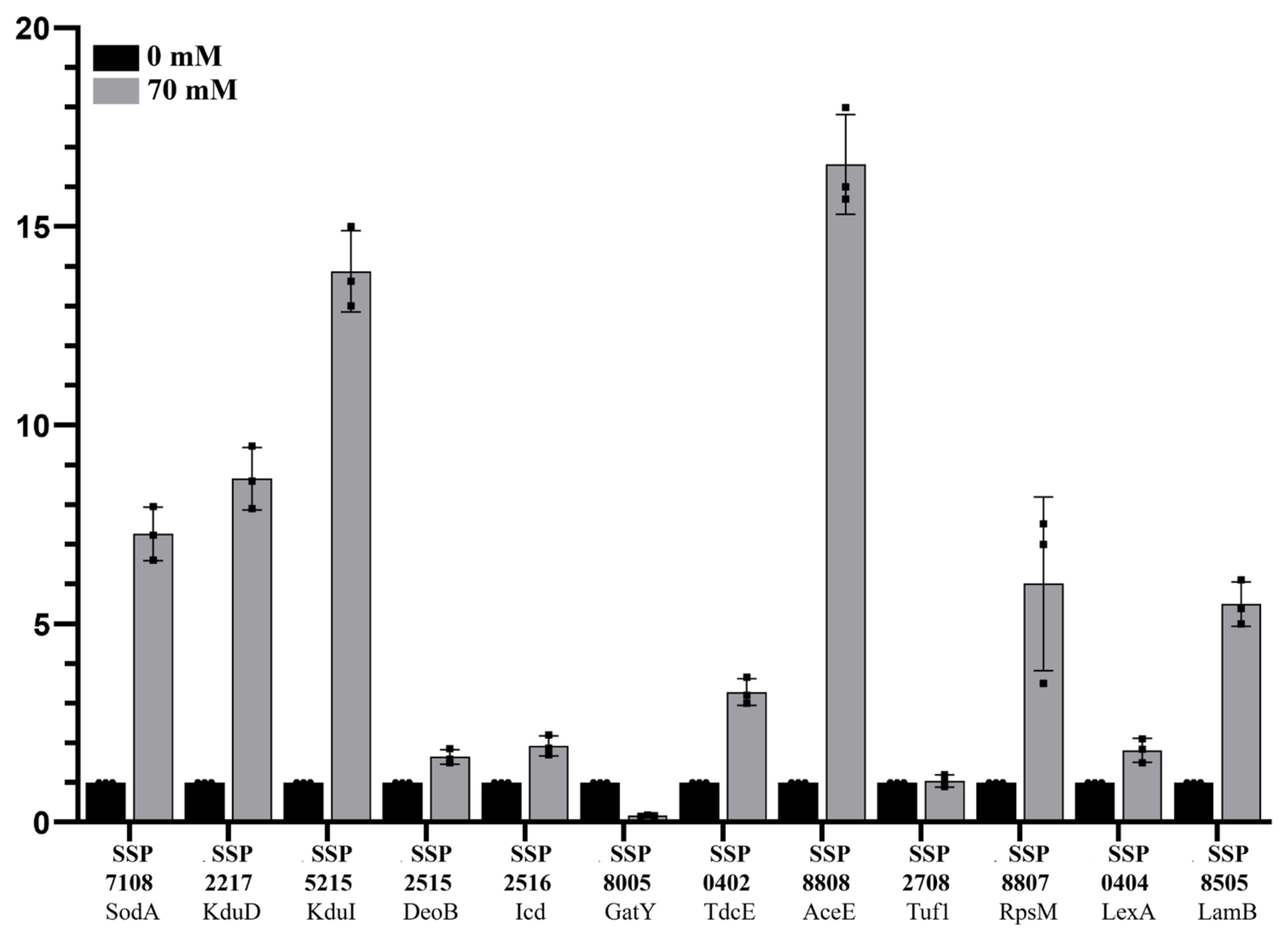

Supplement: Figure S — Comparative bar graph of selected cytoplasmic proteins showing differential expression in E. coli BW25113 under boric acid stress. Bars represent the mean normalized spot volumes (%Vol) of proteins from control (0 mM boric acid, black) and 70 mM boric acid-treated (grey) cultures. Error bars indicate standard deviation (n = 3). The X-axis displays the Standard Spot Numbers (SSP) and protein names of the differentially expressed proteins. The Y-axis represents the relative value of the normalized spot volume (%Vol). This graph was generated using the ImageJ program based on data obtained from 2D-PAGE gels analyzed with PDQuest 8.0.1 software. [file tjb-49-03-280s1.tif]
